# Reduced mRNA expression levels of *NFE2L2* are associated with poor outcome in breast cancer patients

**DOI:** 10.1186/s12885-016-2840-x

**Published:** 2016-10-22

**Authors:** Barbara Wolf, Georg Goebel, Hubert Hackl, Heidi Fiegl

**Affiliations:** 1 Department of Obstetrics and Gynaecology, Medical University of Innsbruck, Anichstr. 35, 6020 Innsbruck, Austria; 2 Department of Medical Statistics, Informatics and Health Economics, Medical University of Innsbruck, 6020 Innsbruck, Austria; 3 Division of Bioinformatics, Biocenter, Medical University of Innsbruck, 6020 Innsbruck, Austria

**Keywords:** Breast cancer, NFE2L2, Biomarker, Prediction, Translational cancer research

## Abstract

**Background:**

The transcription factor nuclear factor erythroid 2-related factor 2 (NFE2L2; previously known as NRF2) is a crucial regulator of the intracellular antioxidant response. It controls the expression of genes involved in the detoxification and elimination of reactive oxidants and electrophilic agents. The role of NFE2L2 in cancer is subject of controversial discussion, as it has been reported to have both pro-and anti-tumourigenic functions. To shed some light on this paradox, we analysed the *NFE2L2* mRNA expression levels in breast cancer and its association with clinicopathological features and survival.

**Methods:**

We retrospectively evaluated the *NFE2L2* mRNA expression levels in tumour tissue of two independent breast cancer patient cohorts. In the training set we analysed data from the Molecular Taxonomy of Breast Cancer International Consortium (METABRIC). In the test set we measured the *NFE2L2* mRNA expression levels in 176 breast tumour tissues by quantitative real-time reverse transcription PCR (qRT-PCR). Group differences were analysed using Mann–Whitney *U*-test, and associations between *NFE2L2* mRNA expression levels and clinicopathological features were examined by means of univariate and multivariate survival analyses. Furthermore, we compared *NFE2L2* mRNA expression levels between tumour and normal breast tissue samples by means of 108 paired samples from the The Cancer Genome Atlas (TCGA) dataset.

**Results:**

In the training set we identified an independent predictive value for high *NFE2L2* mRNA expression levels [HR_disease specific death_ 0.8 (0.6–1.0), *P* = 0.041; HR_death_ 0.8 (0.6–1.0), *P* = 0.023] especially in the subgroup of oestrogen receptor (ER) positive tumours [HR_disease specific death_ 0.6 (0.4–0.9), *P* = 0.008; HR_death_ 0.6 (0.4–0.8), *P* = 0.001]. Similarly, we found this association also in the test set [HR_relapse_ 0.4 (0.2–0.9), *P* = 0.031] and again, more pronounced in patients with ER positive tumours [HR_relapse_ 0.2 (0.1–0.7), *P* = 0.012]. In addition, we observed generally lower NFE2L2 expression levels in tumour tissues than in normal breast tissues.

**Conclusion:**

We concluded that reduced *NFE2L2* mRNA expression in tumour tissues is an independent predictor of shortened survival in breast cancer patients.

## Background

Breast cancer is the most frequent cancer diagnosed in women across the globe, accounting for 25 % of all cancer cases and with an estimated 1.7 million new cases per year worldwide. Moreover, with 15 % of all cancer deaths, breast cancer is still the most common cause for cancer death in women in both developing and developed regions [1]. Further insight into the biology of breast cancer is required and, besides that, additional markers are needed to improve treatment efficiency and patient outcome.

The gene nuclear factor, erythroid 2-like 2 (NFE2L2; previously known as NRF2) encodes a basic leucine zipper (bZIP) transcription factor of the cap’n’collar (CNC) family [2]. NFE2L2 regulates the expression of a subset of genes, including phase II detoxifying enzymes, intracellular redox-balancing proteins and transporters [3–6]. Under physiologic conditions, NFE2L2 is located in the cytoplasm where it is bound by its redox-sensitive adapter protein kelch-like erythroid cell-derived protein with CNC homology (ECH)-associated protein 1 (KEAP1) and cullin 3 (CUL3), the core component of the E3 ubiquitin ligase, which target NFE2L2 for constant proteasomal degradation. In response to NFE2L2 inducers such as excess of reactive oxygen species (ROS) leading to oxidative stress or chemopreventive compounds, KEAP1 undergoes conformational changes that partially disrupt the interaction with NFE2L2. Thus, NFE2L2 is stabilized, accumulates and translocates to the nucleus, where it dimerizes with members of the small musculoaponeurotic fibrosarcoma (MAF) protein family and binds to antioxidant response elements (ARE) or MAF recognition elements (MARE) in the promoter sequence of its target genes to initiate their transcription [7–12].

Various groups reported increased susceptibility to chemically induced carcinogenesis and decreased protection from metastasis in *Nfe2l2*-deficient mice [13–17]. Therefore, NFE2L2 has long been considered a cytoprotective transcription factor which is essential for the defence against oxidative stress and activation of the NFE2L2 pathway has been proposed as potential preventive strategy against carcinogenesis due to its function as a master regulator of the expression of antioxidant and detoxifying enzymes [18, 19]. Interestingly, an increasing number of contrasting findings is emerging, uncovering the’dark side of NFE2L2’ [20, 21]. One research group, for example, reported an association between high NFE2L2 expression and aggressive tumour behaviour [22]. Taken together, it seems that NFE2L2 plays a dual role in cancer.

In the present study we investigated the predictive role of *NFE2L2* mRNA expression levels in breast cancer patients of two independent cohorts. First, we used the publicly available transcriptomic dataset of the Molecular Taxonomy of Breast Cancer International Consortium (METABRIC) with overall survival (OS) and disease-specific survival (DSS) data of 1942 patients as training set and second, a cohort derived from our own biobank consisting of 176 breast cancer patients including OS and relapse-free survival (RFS) data as test set.

## Methods

### Study design, patients and specimens

We retrospectively analysed three independent data sets:

(1) In a first step data from the publicly available METABRIC dataset were used as training set to retrospectively explore *NFE2L2* mRNA expression levels [23] and their predictive association with outcome variables. This dataset includes OS and DSS data as well as gene expression and DNA copy number data from 1981 resected primary breast tumours. We excluded 39 patients who showed either ductal carcinoma in situ (*n* = 10), unknown histological differentiation (*n* = 24) or phyllodes tumours (*n* = 5). Patients with HER2 positive breast cancer did not receive anti-HER2 therapy. The median age at diagnosis was 61.8 years (aged 21.9 to 96.3 years). All clinical and genomic data is publicly available at the European Genome-phenome Archive (EGAS00000000083) [23].

Patient characteristics and clinicopathological features are summarized in Table 1A.

**Table 1:** Association of *NFE2L2* mRNA expression with clinicopathologic features

A			NFE2L2 mRNA expression	
			log_2_ values	
		n	Mean (+/− SD)	*P*
Age	< median age (61.79 years)	971	9.13 (0.56)	**<0.001**
	≥ median age (61.79 years)	971	9.04 (0.60)	
Size	T1	842	9.10 (0.59)	0.200
	T2/3/4	1082	9.07 (0.57)	
	n.a.	18		
LN	negative	1007	9.07 (0.59)	0.213
	positive	929	9.10 (0.58)	
	n.a.	6		
Tumour grade	I	163	9.18 (0.57)	**0.021**
	II	767	9.09 (0.57)	
	III	947	9.07 (0.58)	
	n.a.	65		
Histology	invasive lobular	147	9.09 (0.65)	**0.001**
	invasive ductal	1548	9.06 (0.58)	
	special differentiation	148	9.22 (0.54)	
	lobular and ductal mixed forms	90	9.22 (0.58)	
	only “invasive tumour” as information	9	9.27 (0.33)	
MP	premenopausal	428	9.15 (0.54)	**0.003**
	postmenopausal	1503	9.06 (0.59)	
	n.a.	11		
HER2	score 0/+	667	9.11 (0.51)	**0.041**
	score ++/+++	147	9.19 (0.50)	
	n.a.	1128		
ER	neg	432	9.07 (0.58)	0.981
	pos	1482	9.09 (0.58)	
B			NFE2L2 mRNA expression	
			log_e_ values (norm. to TBP)	
		n	Mean (+/− SD)	*P*
Age	< median age (60.2 years)	88	−0.71 (0.32)	0.519
	≥ median age (60.2 years)	88	−0.69 (0.37)	
Size	T1	68	−0.63 (0.28)	**0.045**
	T2/3/4	108	−0.74 (0.38)	
LN	negative	74	−0.70 (0.35)	0.389
	positive	96	−0.71 (0.35)	
	n.a.	6		
Tumour grade	I	27	−0.71 (0.31)	0.513
	II	115	−0.69 (0.34)	
	III	32	−0.74 (0.40)	
	n.a.	2		
Histology	invasive lobular carcinoma	22	−0.69 (0.47)	0.346
	invasive ductal carcinoma	135	−0.68 (0.30)	
	ductal carcinoma with specific differentiation	19	−0.83 (0.48)	
MP	premenopausal	48	−0.69 (0.29)	0.873
	postmenopausal	128	−0.70 (0.37)	
HER2	score 0/+	133	−0.72 (0.38)	0.798
	score ++/+++	40	−0.66 (0.21)	
	n.a.	3		
ER	neg	49	−0.75 (0.41)	0.215
	pos	127	−0.68 (0.32)	
PR	neg	59	−0.78 (0.42)	**0.040**
	pos	117	−0.66 (0.29)	

Abbreviations: *LN* lymph node status, *MP* menopausal status, *HER2* human epidermal growth factor receptor 2 status, *ER* oestrogen receptor status, *PR* progesterone receptor status; n.a, not available

*p*-values were calculated using non-parametric Mann–Whitney test

Bold values have a significance level of *P* < 0.05

(A) Training set: 1942 breast cancer patients, METABRIC data set. (B) Test set: 176 primary breast cancer patient

(2) Next we analysed the *NFE2L2* mRNA expression levels by quantitative reverse-transcription PCR (qRT-PCR) in prospectively collected fresh frozen tumour tissue samples from 176 patients with primary breast cancer (aged 30.2 to 89.6; median age at diagnosis, 60.2 years) and 10 patients with benign breast diseases (aged 19.8 to 46.0; median age at diagnosis, 37.2 years) treated at our department (Department of Obstetrics and Gynaecology, Medical University of Innsbruck, Austria) between October 1990 and April 2010. All patients were monitored within the outpatient follow-up program of our department. Clinical, pathological and follow-up data were stored in a database according to our hospital’s privacy rules. Since the tissues used in this study are from patients diagnosed between 1990 and 2010 not from all patients a written informed consent is available. But in accordance with the Austrian law, the study was reviewed and approved by the Ethics committee of the Medical University of Innsbruck (reference number: AN2015-0228) and it was conducted in accordance with the Declaration of Helsinki. All samples were anonymized before analysis was performed, to guarantee the protection of privacy.

The study was performed in concordance with the Reporting Recommendations for Tumour Marker Prognostic Studies of the National Cancer Institute (REMARK) [24]. Tumour specimens were prepared and stored as previously described [25]. Oestrogen receptor (ER) status and progesterone receptor (PR) status was identified by immunohistochemistry (IHC).

Neoadjuvant chemotherapy was not administered to the patients included in the study.

All patient characteristics and clinicopathological features are summarized in Table 1B.

(3) Paired *NFE2L2* gene expression data from 108 breast cancer patients (tumour vs. normal tissue) from the publicly available The Cancer Genome Atlas (TCGA) dataset were used [26]. The patients ranged in age from 30.7 to 90 years (mean 57.2 years). Thirty breast cancer patients (27.8 %) had T1 tumours and 78 patients (72.2 %) T2-T4 tumours. Sixty two patients (58.5 %) had positive lymph nodes. Seventy five patients (69.4 %) had oestrogen-receptor positive tumours, 66 patients (61.1 %) progesterone receptor positive tumours and 13 patients (12.7 %) HER2 positive tumours.

### RNA isolation and mRNA expression analysis

Procedures were performed as previously described [25]. Primers and probe for *NFE2L2* [GenBank: NM_006164.4] were designed with Primer Express software, version 2.0. The reaction is specific for isoforms 1, 2 and 3. Forward: 5′-AGC CCA GCA CAT CCA GTC A-3′, Reverse: 5′-CAG TCA TCA AAG TAC AAA GCA TCT GA-3′, TaqMan Probe: 5′-FAM- CCA ACT ACT CCC AGG TTG CCC AC-TAMRA-3′.

Primers and probe for the TATA box-binding protein (TBP; endogenous RNA-control) were used according to Bieche et al. [27]. All reactions were obtained from Metabion (Planegg, Germany) and checked if they are specific for mRNA and do not amplify genomic DNA.

### Statistical analysis

The non-parametric Mann–Whitney *U* test was applied in order to compare *NFE2L2* mRNA expression levels between groups.

Overall survival (OS) was defined as the time from surgery to death from any cause or to the last clinical inspection, and disease-specific survival (DSS) as the time from surgery to breast cancer specific death. Relapse-free survival (RFS) was defined as the time from surgery to histo-pathological confirmation of distant metastases or regional recurrence.

Univariate Kaplan-Meier analyses and multivariate Cox survival analyses were used to explore the association of *NFE2L2* mRNA expression levels with RFS, OS and DSS.

First, univariate Kaplan-Meier curves for tumour size, lymph node status, grade, tumour histology, menopausal status, HER2 and ER status, the application of chemotherapy, radiation therapy or endocrine therapy and *NFE2L2* mRNA expression were calculated using the log-rank test to compare the survival distributions between groups. For survival analysis, *NFE2L2* mRNA expression levels were dichotomized into low and high using the 65^th^ percentile expression value, which was identified as the optimal threshold in the training set using Youden’s index [28] based on a receiver operating characteristic (ROC) curve analysis.

Second, we used a time-independent Cox-proportional hazard approach for multivariate survival analysis to estimate hazard ratios (HR) and 95 % Confidence interval (CI).

For the comparison of *NFE2L2* mRNA expression in paired samples (normal and breast cancer tissues) from 108 breast cancer patients the Wilcoxon paired-sample test was applied. Statistical analysis was performed using SPSS statistical software (version 20.0; SPSS Inc., Chicago, IL, USA).

## Results

### *NFE2L2* mRNA expression levels and clinicopathological features in breast cancer patients

In the training set (METABRIC dataset, consisting of 1942 patients) we identified associations between *NFE2L2* mRNA expression levels and the patient’s age (*P* < 0.001), tumour histology (*P* = 0.001), menopausal status (*P* = 0.003) and HER2 status (*P* = 0.041) (Table 1A). But none of these findings could be validated in our test set consisting of 176 breast tumour tissues from the local biobank at the Department of Obstetrics and Gynaecology, Medical University of Innsbruck (Table 1B). However, we could observe significantly higher *NFE2L2* mRNA expression levels in smaller tumours (T1) compared to larger ones (T2/3/4) (*P* = 0.045), and in progesterone receptor (PR) positive tumours (Table 1B; *P* = 0.040).

### *NFE2L2* mRNA expression levels and survival of breast cancer patients

**Fig. 1:**
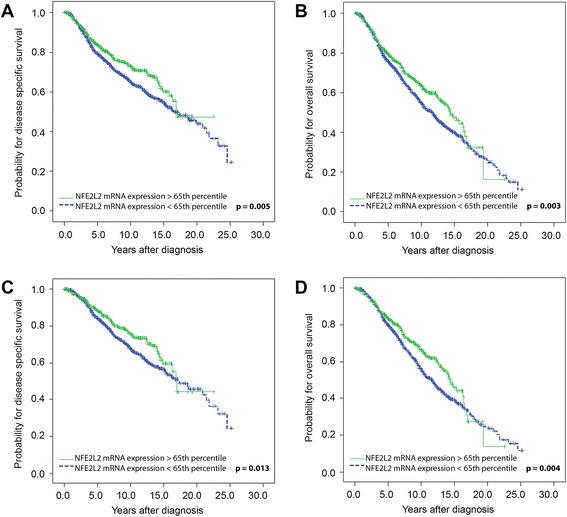
Kaplan Meier survival analysis and *NFE2L2* mRNA expression in the training set. **(a)** Disease specific survival and (**b**) Overall survival in 1942 breast cancer patients. **(c)** Disease specific survival and (**d**) Overall survival in oestrogen receptor positive breast tumours from 1482 patients

In the training cohort we identified the 65^th^ percentile regarding *NFE2L2* mRNA expression levels as an optimal cut-off value to discriminate between breast cancer patients with a better DSS and those with a poorer outcome. Univariate survival analysis of all 1942 breast cancer patients revealed that patients with high *NFE2L2* mRNA expression levels had a better DSS (*P* = 0.005) and OS (*P* = 0.003) in comparison to those with low *NFE2L2* mRNA expression levels (Table 2; Fig. 1a, b).

**Table 2:** Univariate survival analysis

A. Variable		Disease specific survival		Overall survival	
		No.Patients	*P*	No.Patients	*P*
		(died/total)	(logrank-Test)	(died/total)	(logrank-Test)
Size	T1	198/842	**<0.001**	307/842	**<0.001**
	T2/3	414/1082		560/1082	
LN	negative	234/1007	**<0.001**	386/1007	**<0.001**
	positive	383/929		488/929	
Tumour grade	I	27/163	**<0.001**	50/163	**<0.001**
	II	208/767		315/767	
	III	365/947		473/947	
Histology	invasive lobular	48/147	**<0.001**	69/147	**<0.001**
	invasive ductal	520/1548		727/1548	
	special differentiation	20/148		41/148	
	lobular and ductal mixed forms	27/90		36/90	
	only “invasive tumour” as information	4/9		4/9	
MP	premenopausal	141/428	0.877	152/428	**<0.001**
	postmenopausal	472/1503		719/1503	
HER2	neg	199/667	**<0.001**	287/667	**<0.001**
	pos	61/147		73/147	
ER	neg	183/432	**<0.001**	213/432	**0.001**
	pos	429/1482		653/1482	
Chemotherapy	no	433/1526	**<0.001**	682/1526	**<0.001**
	yes	186/416		195/416	
Radiation therapy	no	265/781	0.461	396/781	**0.028**
	yes	354/1161		481/1161	
Endocrine therapy	no	255/733	0.820	343/733	**0.024**
	yes	364/1209		534/1209	
*NFE2L2* mRNA expression	low (<65^th^ %ile)	453/1268	**0.005**	641/1268	**0.003**
	high (>65^th^ %ile)	166/674		236/674	
*NFE2L2* mRNA expression in ER pos tumours	low (<65^th^ %ile)	318/968	**0.013**	485/968	**0.004**
	high (>65^th^ %tile)	111/514		168/514	
B. Variable		Relapse-free survival		Overall survival	
		No.Patients	*P*	No.Patients	*P*
		(relapsed/total)	(logrank-Test)	(died/total)	(logrank-Test)
Size	T1	12/68	**0.022**	17/68	**0.006**
	T2/3/4	37/108		56/108	
LN	negative	14/74	**0.026**	22/74	**0.003**
	positive	33/96		47/96	
Tumour grade	I	3/27	0.154	15/27	0.764
	II	34/115		42/115	
	III	12/32		15/32	
Histology	invasive lobular carcinoma	5/22	0.288	5/22	0.487
	invasive ductal carcinoma	41/135		59/135	
	ductal carcinoma with specific differentiation	3/19		9/19	
MP	premenopausal	14/48	0.970	14/48	**0.032**
	postmenopausal	35/128		59/128	
HER2	neg	36/133	0.971	51/133	0.692
	pos	12/40		20/40	
ER	neg	20/49	0.061	26/49	0.831
	pos	29/127		47/127	
PR	neg	24/59	**0.021**	32/59	0.234
	pos	25/117		41/117	
Chemotherapy	no	18/90	**0.022**	35/90	0.382
	yes	31/86		38/86	
Radiation therapy	no	14/68	0.348	33/68	0.069
	yes	35/107		40/107	
Endocrine therapy	no	18/51	0.232	30/51	0.433
	yes	31/125		43/125	
*NFE2L2* mRNA expression	low (<65^th^ %ile)	39/114	**0.013**	55/114	0.081
	high (>65^th^ %ile)	10/62		18/62	
	low (< median)	36/88	**<0.001**	48/88	**0.004**
	high (> median)	13/88		25/88	
*NFE2L2* mRNA expression in ER pos tumours	low (<65^th^ %ile)	25/82	**0.005**	37/82	**0.034**
	high (>65^th^ %ile)	4/45		10/45	
	low (< median)	23/59	**<0.001**	32/59	**<0.001**
	high (> median)	6/68		15/68	

(A) Disease specific and overall survival in 1942 breast cancer patients in the METABRIC dataset. (B) Relapse-free and overall survival in 176 patients with primary breast cancer

Bold values have a significance level of *P* < 0.05

Since the NFE2L2 pathway was recently shown to be more active in steroid receptor positive breast cancer [29], we focused on ER status. Interestingly, we identified the prognostically relevant association between high *NFE2L2* mRNA expression levels and better DSS and OS in the subgroup of patients with ER positive tumours (DSS: *P* = 0.013; OS: *P* = 0.004; Table 2A; Fig. 1c, d), but not in patients with ER negative tumours.

In the multivariate Cox-regression analysis high *NFE2L2* mRNA expression levels have been validated as a marker with independent, predictive value for a reduced risk for disease specific death or death from any cause in the whole cohort [HR_disease specific death_ 0.8 (0.6–1.0); *P* = 0.041, HR_death_ 0.8 (0.6–1.0); *P* = 0.023] (Table 3A) and in the ER-positive tumour group [HR_disease specific death_ 0.6 (0.4–0.9); *P* = 0.008, HR_death_ 0.6 (0.4–0.8); *P* = 0.001] (Table 4A).

**Table 3:** Multivariate Cox-regression survival analysis

A		Disease specific survival				Overall survival			
		Regression model without NFE2L2 mRNA expression		Regression model incl. NFE2L2 mRNA expression		Regression model without NFE2L2 mRNA expression		Regression model incl. NFE2L2 mRNA expression	
Variable		HR (95 % CI)	*P*	HR (95 % CI)	*P*	HR (95 % CI)	*P*	HR (95 % CI)	*P*
Size	T1 vs. T2/3/4	**1.5 (1.2**–**2.0)**	**0.003**	**1.5 (1.2**–**2.0)**	**0.002**	**1.5 (1.2**–**1.8)**	**0.001**	**1.5 (1.2**–**1.9)**	**0.001**
LN	neg. vs. pos.	**1.7 (1.2**–**2.3)**	**0.002**	**1.7 (1.2**–**2.4)**	**0.001**	**1.5 (1.2**–**2.0)**	**0.002**	**1.6 (1.2**–**2.1)**	**0.001**
Tumour grade	I vs. II vs. III	**1.4 (1.1 -1.9)**	**0.008**	**1.4 (1.1 -1.8)**	**0.014**	**1.3 (1.0 -1.6)**	**0.026**	**1.3 (1.0 -1.6)**	**0.043**
MP	pre vs. post	1.3 (0.9–1.7)	0.139	1.2 (0.9–1.7)	0.175	**1.7 (1.2**–**2.2)**	**<0.001**	**1.6 (1.2**–**2.1)**	**0.001**
HER2	neg. vs. pos.	**1.6 (1.2**–**2.2)**	**0.002**	**1.7 (1.2**–**2.2)**	**0.001**	**1.5 (1.1**–**2.0)**	**0.004**	**1.5 (1.2**–**2.0)**	**0.002**
ER	neg. vs. pos.	1.2 (0.8–1.8)	0.432	1.2 (0.8–1.7)	0.466	1.1 (0.8–1.6)	0.449	1.1 (0.8–1.6)	0.496
Histology		0.9 (0.7–1.2)	0.422	0.9 (0.7–1.2)	0.462	1.0 (0.8–1.2)	0.641	1.0 (0.8–1.2)	0.695
Chemotherapy	no vs. yes	**1.7 (1.1**–**2.5)**	**0.014**	**1.7 (1.1**–**2.5)**	**0.015**	**1.5 (1.0**–**2.2)**	**0.027**	**1.5 (1.0**–**2.2)**	**0.030**
Radiation therapy	no vs. yes	1.0 (0.7–1.3)	0.955	1.0 (0.7–1.3)	0.850	0.9 (0.7–1.1)	0.298	0.9 (0.7–1.1)	0.232
Endocrine therapy	no vs. yes	0.8 (0.6–1.1)	0.211	0.8 (0.6–1.1)	0.232	0.8 (0.6–1.1)	0.214	0.8 (0.6–1.1)	0.253
*NFE2L2* mRNA expression	low vs. high (< or > 65^th^ %ile)	-	-	**0.8 (0.6**–**1.0)**	**0.041**	-	-	**0.8 (0.6**–**1.0)**	**0.023**
B		Relapse-free survival				Overall survival			
		Regression model without NFE2L2 mRNA expression		Regression model incl. NFE2L2 mRNA expression		Regression model without NFE2L2 mRNA expression		Regression model incl. NFE2L2 mRNA expression	
Variable		HR (95 % CI)	*P*	HR (95 % CI)	*P*	HR (95 % CI)	*P*	HR (95 % CI)	*P*
Size	T1 vs. T2/3/4	1.9 (0.8–4.3)	0.120	2.0 (0.9–4.5)	0.098	1.6 (0.8–3.2)	0.176	1.6 (0.8–3.2)	0.154
LN	neg. vs. pos.	1.4 (0.7–2.9)	0.368	1.3 (0.6–2.7)	0.539	**2.2 (1.2**–**4.3)**	**0.015**	**2.1 (1.1**–**4.1)**	**0.025**
Tumour grade	I vs. II vs. III	1.1 (0.6–2.1)	0.709	1.3 (0.7–2.5)	0.378	0.8 (0.5–1.2)	0.257	0.8 (0.5–1.4)	0.455
MP	pre vs. post	1.0 (0.5–2.1)	0.892	1.1 (0.5–2.2)	0.817	**2.0 (1.0**–**4.0)**	**0.044**	**2.0 (1.0**–**4.0)**	**0.038**
HER2	neg. vs. pos.	1.0 (0.5–2.0)	0.995	1.0 (0.5–2.0)	0.995	1.3 (0.8–2.3)	0.342	1.4 (0.8–2.4)	0.292
ER	neg. vs. pos.	0.5 (0.1–1.7)	0.257	0.4 (0.1–1.5)	0.177	1.0 (0.4–2.8)	0.990	0.9 (0.3–2.5)	0.824
PR	neg. vs. pos.	1.0 (0.4–2.8)	0.992	1.2 (0.4–3.6)	0.726	1.0 (0.4–2.5)	0.982	1.1 (0.5–2.9)	0.782
Histology		0.7 (0.4–1.3)	0.270	0.6 (0.3–1.1)	0.107	1.2 (0.7–2.1)	0.533	1.2 (0.6–2.1)	0.659
Chemotherapy	no vs. yes	1.4 (0.7–3.0)	0.366	1.3 (0.6–2.8)	0.495	0.9 (0.5–1.7)	0.831	0.9 (0.5–1.6)	0.684
Radiation therapy	no vs. yes	1.6 (0.8–3.1)	0.202	1.5 (0.7–2.9)	0.277	0.8 (0.5–1.3)	0.374	0.8 (0.4–1.3)	0.308
Endocrine therapy	no vs. yes	1.2 (0.5–3.0)	0.731	1.3 (0.5–3.4)	0.563	0.7 (0.3–1.5)	0.358	0.7 (0.4–1.5)	0.431
*NFE2L2* mRNA expression	low vs. high (< or > 65^th^ %ile)	-	-	**0.4 (0.2**–**0.9)**	**0.031**	-	-	0.6 (0.3–1.2)	0.135
	*low* vs. *high (< or > median)*	*-*	*-*	***0.3 (0.1***–***0.6)***	***0.001***	*-*	*-*	***0.5 (0.3***–***0.8)***	***0.010***

Abbreviations: *LN* lymph node status, *MP* menopausal status, *HER2* human epidermal growth factor receptor 2 status, *ER* oestrogen receptor status, *PR* progesterone receptor status, *HR* hazard ratio

(A) Disease specific survival and overall survival in 1942 breast cancer patients (METABRIC dataset). (B) Relapse-free survival and overall survival in 176 patients with primary breast cancer

Bold values have a significance level of *P* < 0.05

**Table 4:** Multivariate Cox-regression survival analysis in patients with ER pos. breast cancer

A		Disease specific survival				Overall survival			
		Regression model without NFE2L2 mRNA expression		Regression model incl. NFE2L2 mRNA expression		Regression model without NFE2L2 mRNA expression		Regression model incl. NFE2L2 mRNA expression	
Variable		HR (95 % CI)	*P*	HR (95 % CI)	*P*	HR (95 % CI)	*P*	HR (95 % CI)	*P*
Size	T1 vs. T2/3/4	**1.8 (1.3**–**2.5)**	**0.001**	**1.8 (1.3**–**2.5)**	**0.001**	**1.6 (1.2**–**2.1)**	**0.001**	**1.6 (1.2**–**2.2)**	**0.001**
LN	neg. vs. pos.	**1.9 (1.3**–**2.8)**	**0.001**	**2.0 (1.3**–**2.9)**	**0.001**	**1.6 (1.2**–**2.3)**	**0.002**	**1.7 (1.3**–**2.4)**	**0.001**
Tumour grade	I vs. II vs. III	**1.5 (1.2**–**2.0)**	**0.004**	**1.5 (1.1 -2.0)**	**0.006**	**1.3 (1.1 -1.7)**	**0.013**	**1.3 (1.0 -1.6)**	**0.024**
MP	pre vs. post	1.4 (0.9–2.2)	0.094	1.3 (0.9–2.1)	0.177	**1.9 (1.3**–**2.8)**	**0.001**	**1.7 (1.2**–**2.6)**	**0.006**
HER2	neg. vs. pos.	1.5 (1.0–2.3)	0.074	1.5 (1.0–2.3)	0.058	1.4 (1.0–2.0)	0.084	1.4 (1.0–2.1)	0.062
Histology		1.0 (0.7–1.3)	0.870	1.0 (0.8–1.3)	0.981	1.0 (0.8–1.2)	0.987	1.0 (0.8–1.3)	0.876
Chemotherapy	no vs. yes	**2.0 (1.2**–**3.4)**	**0.005**	**2.0 (1.2**–**3.4)**	**0.006**	**1.8 (1.1**–**2.9)**	**0.013**	**1.8 (1.1**–**2.9)**	**0.013**
Radiation therapy	no vs. yes	1.0 (0.7–1.4)	0.994	1.0 (0.7–1.3)	0.832	0.9 (0.7–1.1)	0.242	0.8 (0.6–1.1)	0.141
Endocrine therapy	no vs. yes	0.7 (0.5–1.2)	0.192	0.8 (0.5–1.2)	0.297	0.8 (0.6–1.2)	0.237	0.9 (0.6–1.2)	0.392
*NFE2L2* mRNA expression	low vs. high (< or > 65^th^ %ile)	-	-	**0.6 (0.4**–**0.9)**	**0.008**	-	-	**0.6 (0.4**–**0.8)**	**0.001**
B		Relapse-free survival				Overall survival			
		Regression model without NFE2L2 mRNA expression		Regression model incl. NFE2L2 mRNA expression		Regression model without NFE2L2 mRNA expression		Regression model incl. NFE2L2 mRNA expression	
Variable		HR (95 % CI)	*P*	HR (95 % CI)	*P*	HR (95 % CI)	*P*	HR (95 % CI)	*P*
Size	T1 vs. T2/3/4	2.6 (1.0–6.9)	0.056	**2.9 (1.1**–**8.0)**	**0.038**	1.8 (0.8–3.9)	0.152	1.9 (0.8–4.1)	0.128
LN	neg. vs. pos.	1.7 (0.7–4.6)	0.255	1.7 (0.6–4.6)	0.289	**3.1 (1.3**–**7.3)**	**0.008**	**2.8 (1.2**–**6.5)**	**0.016**
Tumour grade	I vs. II vs. III	0.9 (0.4–2.4)	0.908	1.6 (0.5–4.6)	0.403	0.6 (0.3–1.2)	0.122	0.7 (0.3–1.4)	0.313
MP	pre vs. post	0.6 (0.2–1.4)	0.221	0.5 (0.2–1.1)	0.086	1.1 (0.5–2.7)	0.756	1.1 (0.5–2.6)	0.778
HER2	neg. vs. pos.	0.4 (0.1–1.2)	0.107	0.4 (0.1–1.1)	0.079	0.7 (0.3–1.5)	0.338	0.7 (0.3–1.6)	0.420
Histology		1.5 (0.6–3.7)	0.375	1.1 (0.4–2.8)	0.895	**2.1 (1.0**–**4.4)**	**0.039**	**2.2 (1.0**–**4.6)**	**0.045**
Chemotherapy	no vs. yes	0.9 (0.3–2.2)	0.761	0.7 (0.3–1.8)	0.484	0.7 (0.3–1.6)	0.437	0.7 (0.3–1.5)	0.296
Radiation therapy	no vs. yes	1.7 (0.7–4.0)	0.250	1.5 (0.6–3.6)	0.349	0.7 (0.3–1.3)	0.209	0.7 (0.3–1.3)	0.211
Endocrine therapy	no vs. yes	0.9 (0.2–3.4)	0.879	1.4 (0.4–5.6)	0.616	1.0 (0.3–2.8)	0.986	1.1 (0.4–3.2)	0.823
*NFE2L2* mRNA expression	low vs. high (< or > 65^th^ %ile)	-	-	**0.2 (0.1**–**0.7)**	**0.012**	-	-	0.5 (0.2–1.1)	0.072
	*low* vs. *high (< or > median)*	*-*	*-*	***0.2 (0.1***–***0.5)***	***0.001***	*-*	*-*	***0.4 (0.2***–***0.8)***	***0.009***

*Abbreviations*: *LN* lymph node status, *MP* menopausal status, *HER2* human epidermal growth factor receptor 2 status, *HR* hazard ratio

(A) Disease specific and overall survival in 1482 patients (METABRIC dataset). (B) Relapse-free and overall survival in 127 patients

Bold values have a significance level of *P* < 0.05

### Validation of associations between *NFE2L2* mRNA expression levels and survival of breast cancer patients

**Fig. 2:**
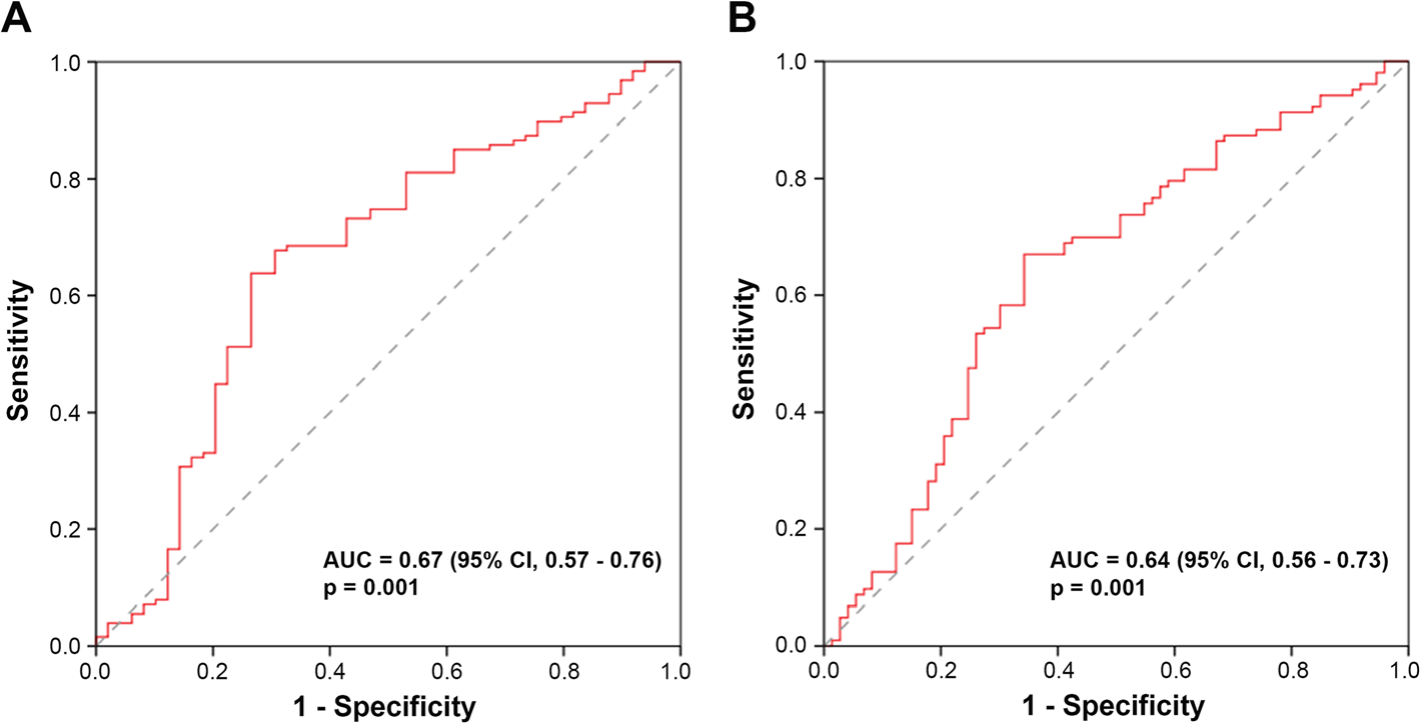
Receiver operating characteristic (ROC) curve analysis and *NFE2L2* mRNA expression in the test set. (**a**) Relapse-free survival and (**b**) overall survival in 176 breast cancer patients

To validate the identified association of high *NFE2L2* mRNA expression levels with a favourable patient outcome within an independent cohort we analysed 176 breast tumour tissue samples from our local biobank. The ROC curves in Fig. 2 show the statistically significant ability of *NFE2L2* mRNA expression to be used as a prognostic marker to predict the likelihood of disease recurrence with an area under the curve (AUC) value of 0.67 (95 % CI, 0.57 – 0.76; *p* = 0.001) or of death with an AUC-value of 0.64 (95 % CI; 0.56 – 0.73). As cut-off value for the discrimination of *NFE2L2* high and low mRNA expression levels we consistently applied the 65^th^ percentile, as identified by means of the training set. Additionally, we analysed the data on the basis of the often used median value as cut-off value.

**Fig. 3:**
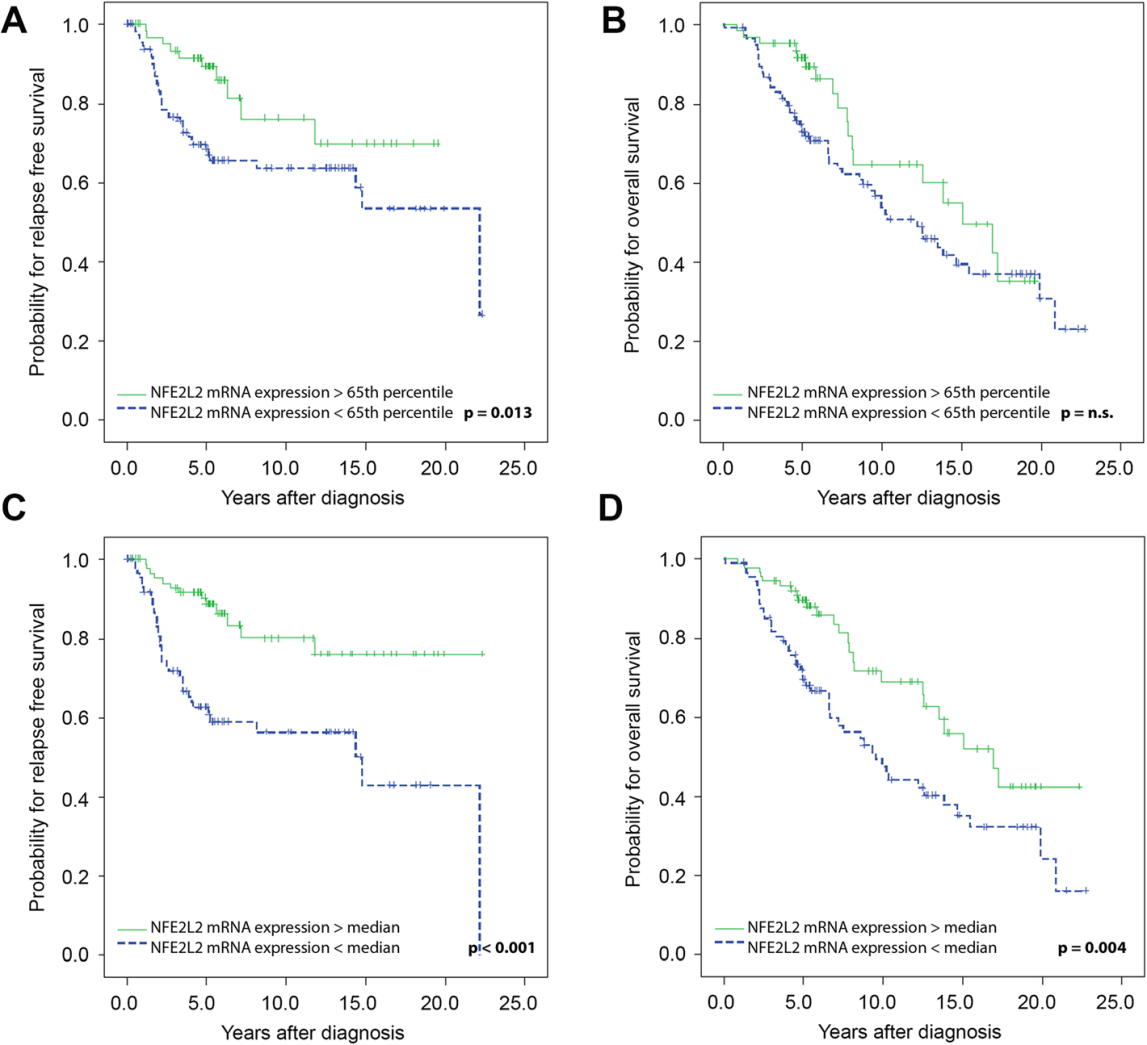
Kaplan Meier survival analysis and *NFE2L2* mRNA expression in the test set. (**a**) Relapse-free survival and (**b**) overall survival in 176 breast cancer patients according the 65^th^ percentile as cut-off value as identified by Youden’s index. (**c**) Relapse-free survival and (**d**) overall survival in 176 breast cancer patients according the median as cut-off value

**Fig. 4:**
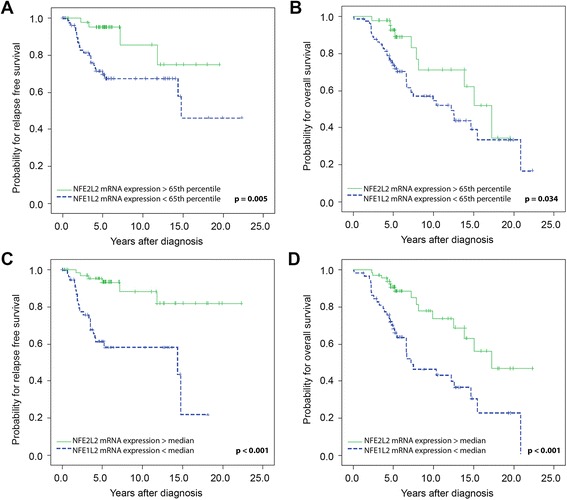
Kaplan Meier survival analysis and *NFE2L2* mRNA expression in 127 patients with oestrogen receptor positive breast cancer of the test set. (**a**) Relapse-free survival and (**b**) overall survival according the 65^th^ percentile as cut-off value. (**c **) Relapse-free survival and (**d**) overall survival in oestrogen receptor positive breast tumours from 127 patients according the median as cut-off value

Univariate survival analysis of all 176 breast cancer patients composing the test set using the 65^th^ percentile as cut-off revealed that breast cancer patients with high *NFE2L2* mRNA expression levels had a better RFS (*P* = 0.013) in comparison to those with low *NFE2L2* mRNA expression levels. However, there was no statistically relevant difference regarding OS (Table 2B; Fig. 3a, bs). Using the median as cut-off value, patients with high *NFE2L2* mRNA expression levels had a better RFS (*P* < 0.001) and also OS (*P* = 0.004) compared to those with low *NFE2L2* mRNA expression levels (Table 2B; Fig. 3c, d). The results of the subgroup analysis of 127 patients with ER positive tumours validated those obtained from the training set for RFS and OS for both cut-off types (65^th^ percentile: RFS: *P* = 0.005; OS: *P* = 0.034; median: RFS: *P* < 0.001; OS: *P* < 0.001) (Table 2B; Fig. 4). Similar findings were observed in the subgroup analysis of 117 patients with PR positive tumours for RFS and OS for both cut-off types (65^th^ percentile: RFS: *P* = 0.030; OS: *P* = 0.035; median: RFS: *P* = 0.002; OS: *P* = 0.008) (data not shown).

Moreover, high *NFE2L2* mRNA expression levels, as defined by the 65^th^ percentile value, remained the strongest independent factor for a reduced risk of relapse in the Cox regression model [HR_relapse_ 0.4 (0.2–0.9), *P* = 0.031] (Table 3B), as well as in the subgroup of patients with ER positive tumours [HR_relapse_ 0.2 (0.1–0.7), *P* = 0.012] (Table 4B).

Applying the median value as cut-off, *NFE2L2* mRNA expression levels remained the strongest factor predicting the risk for relapse and death in the whole cohort [HR_relapse_ 0.3 (0.1–0.6); *P* = 0.001, HR_death_ 0.5 (0.3–0.8); *P* = 0.010] (Table 3B) as well as in the ER positive tumour subgroup [HR_relapse_ 0.2 (0.1–0.5), *P* = 0.001; HR_death_ 0.4 (0.2–0.8), *P* = 0.009] (Table 4B). Similar findings were observed for RFS in the subgroup analysis of 117 patients with PR positive tumours [HR_relapse_ 0.3 (0.1–0.8); *P* = 0.020] (data not shown).

### Comparison of *NFE2L2* mRNA expression levels between tumour and normal breast tissues

**Fig. 5:**
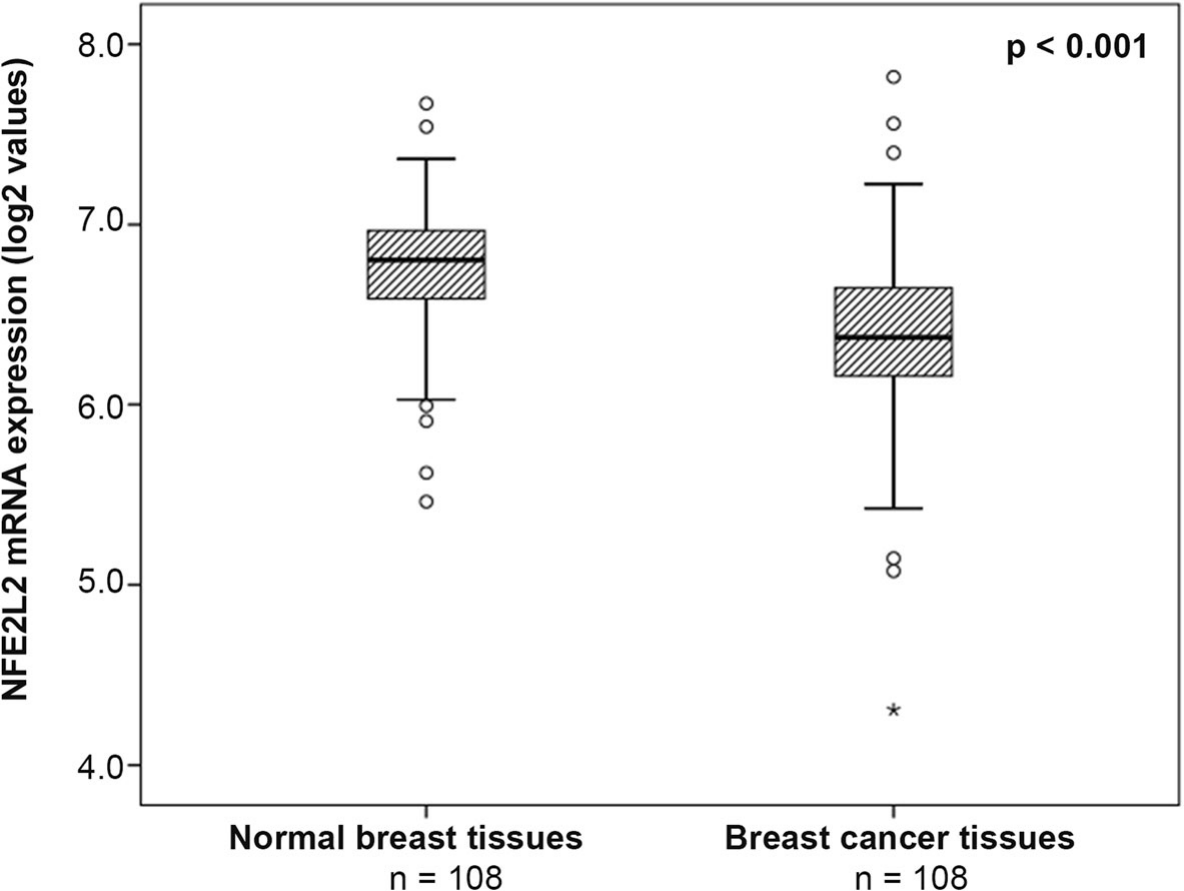
Analysis of paired breast tissue samples. Comparison of *NFE2L2* mRNA expression levels between paired data from tumour tissue and respective normal tissue from 108 breast cancer patients (TCGA dataset). Mean values and standard deviations are depicted in the diagram. Outliers are indicated by circles, extreme values by asterisks. The Wilcoxon paired-sample test was applied to compute the *p*-value.

We compared *NFE2L2* mRNA expression levels between cancerous and the respective normal breast tissues from a subgroup of 108 breast cancer patients of the TCGA dataset. The analysis of these samples revealed that *NFE2L2* mRNA was significantly higher expressed in normal breast tissue compared to breast tumor tissue of the same patient (*p* < 0.001, Fig. 5).

## Discussion

There are numerous studies reporting NFE2L2 activation in various types of cancer and other diseases (as extensively reviewed in [30–34]). However, there is only little documented about the situation in breast cancer. In the current study we identified a significant beneficial role of elevated *NFE2L2* mRNA expression levels in the tumour for the survival of breast cancer patients based on two independent cohorts in agreement with NFE2L2 acting as a tumour suppressor. This association was even more pronounced in the subgroup of patients with ER positive tumours.

The comparison of paired normal and cancerous breast tissues from 108 breast cancer patients identified a higher *NFE2L2* mRNA expression in normal tissues, what underscores its role as a tumour suppressor. It was not analysed within this study if the downregulation of *NFE2L2* mRNA expression in breast cancer is related to oncogenic *NFE2L2* mutations. Therefore this mechanism cannot be ruled out. But recently Kim et al. analysed 1145 cancer tissues from different carcinomas including breast cancer. They detected NRF2 mutations in oesophagus (8/70; 11.4 %), skin (1/17; 6.3 %), lung (10/125; 8.0 %), and larynx (3/23; 13.0 %) cancers, but not in breast cancer (0/95; 0 %) [35]. Therefore we assume that *NFE2L2* mutations do not play a major role in breast cancer.

Due to the nature of the *NFE2L2* mRNA expression values, the figures of the two independent cohorts can only be compared based on a relative, non-parametric manner. Using Youden’s method in the training set the 65^th^ percentile of *NFE2L2* mRNA expression values was found as an optimal threshold. In the validation set the 65^th^ percentile was confirmed as significant cut-off for relapse free survival in the univariate and the multivariable analysis. For the overall survival the 65^th^ percentile was validated only in the univariate analysis in the subgroup of ER positive tumours. Extended additional analysis showed a slightly better discrimination of the median within the validation dataset, what corresponds to the ROC-analysis indicating a continuous predictive relevance of NFE2L2.

Since NFE2L2 is a transcription factor that is activated as a consequence to oxidative and electrophilic stress, it regulates the transcription of more than 100 genes whose expression subsequently induces an antioxidant response. Assumingly, this response might be advantageous for survival of breast cancer patients. However, high mRNA levels do not always have to result in increased protein levels and thus functional effects. We cannot exclude, whether upregulation of *NFE2L2* expression is only a transcriptional (side-) effect not being further translated into protein or not exerting any cellular functions. Nevertheless, our data indicate a predictive relevance of *NFE2L2* mRNA expression levels in breast tumour tissue for patient survival.

Although NFE2L2 is commonly known as tumour suppressor whose activation protects from cellular insults upon oxidative or electrophilic stress and is thus anti-tumourigenic and promotes cell survival of normal as well as pre-malignant cells, there is accumulating evidence for the’dark side of NFE2L2’: constitutive activation of NFE2L2 enhances survival, progression as well as chemo- and radioresistance also in cancer cells and thus potentially acting as an oncogene under certain circumstances. This dual role of NFE2L2 is extensively discussed and there is common agreement that the arguments of both sides of this paradox are of value and about the great importance of the context [30, 33, 34]. As already mentioned above, there are many reported cancer cases with high NFE2L2 expression, some of which are associated with increased tumourigenesis [36, 37] and therapy resistance [38–43] and a few correlated with poor survival [22, 44, 45]. Indeed, Kawasaki et al. showed poorer OS in gastric cancer upon high NFE2L2 protein expression, but this has not been confirmed as an independent prognostic factor [22].

Contrasting with these observations, a recent study demonstrated that lower *NFE2L2* expression is associated with poorer outcome in cancer using datasets obtained from the TCGA and GEO databases [46]. This report is in line with our findings in breast cancer. Given that these databases provide big data sets consisting of several hundreds of patients, similar to the METABRIC database we used as training set, the statistical power of the survival analyses is high. Buffa et al. described NFE2L2 as a predicted target of miR-144 [47] and observed that patients who had tumours with low miR-144 and high *NFE2L2* mRNA, but also protein expression levels, had an improved distant relapse-free survival, whereas the opposite expression pattern was associated with a poor outcome. This finding further supports our hypothesis that *NFE2L2* mRNA expression level might serve as a predictive marker. However, contrary to our observations, they have identified this association only in 50 patients with ER negative breast tumours.

Interestingly, another study describes a correlation between a more active NFE2L2 pathway and a more favourable outcome in ER/PR positive breast cancer compared to triple negative breast cancer [29], similar to our results found in the ER positive subgroup of breast cancer patients. It has been shown that oestrogen levels in ER-positive tumours are higher than in ER-negative ones [48]. In light of oestrogens acting as important ROS inducers ER positive tumours might accelerate their antioxidant response by upregulation of NFE2L2 activity to limit their exposure to oxidative stress [49]. Wu et al. reported recently that oestrogen can increase Nrf2 activity through activation of the PI3K/Akt/GSK3β pathway in human breast cancer cells [50]. They suggested that hormonal regulation of Nrf2 activity in breast cancer may be an important consideration during various stages of treatment and long-term patient care [50].

As mentioned before, recent studies have shown that the context of NFE2L2 expression has a major influence on whether NFE2L2 exerts tumour suppressive or oncogenic functions. In particular, besides the health status of a cell, the function and the impact of NFE2L2 in tumourigenesis is also affected by: intracellular location of NFE2L2, capability to be inhibited by KEAP1, choice of a small MAF protein as dimerizing partner and genetic polymorphisms leading to altered regulation of *NFE2L2* transcription [51–53]. Additionally, there are cross talks between NFE2L2 signalling and other prominent signalling pathways, such as NF-κB, p53 and Notch1, affecting cell survival and other aspects of cell fate as summarized in a detailed review [54]. These findings add more complexity to the question, whether NFE2L2 protects from or promotes carcinogenesis, while explaining, at least in part, why this issue raises conflicting results and is discussed controversially.

The strength of this study is the analysis of *NFE2L2* mRNA expression levels in two independent cohorts consisting of 2118 patients in total. As a limitation of our study, as already mentioned, the *NFE2L2* mRNA expression levels may not reflect the functionally active protein levels, which could affect the interpretation of these data; however, without repercussions on the major finding concerning the predictive value of *NFE2L2* mRNA expression. Further mRNA but also protein expression studies are needed to validate the results of the present study.

## Conclusions

In summary, in our data we identified a predictive potential of *NFE2L2* mRNA expression levels in breast cancer, especially in ER positive breast cancer, since high *NFE2L2* expression was associated with better survival. Thus, determination of the *NFE2L2* mRNA expression level might be clinically useful to improve the characterization of breast cancer, eventually leading to more efficient and personalized treatment of breast cancer patients.

## Abbreviations

CI: Confidence interval
DSS: Disease specific survival
ER: Oestrogen receptor
HER2: Human epidermal growth factor receptor 2
HR: Hazard ratio
OS: Overall survival
qRT-PCR: Quantitative reverse-transcription PCR
REMARK: Recommendations for Tumour Marker Prognostic Studies of the National Cancer Institute
RFS: Relapse-free survival
ROS: Reactive oxygen species
TBP: TATA box-binding protein

## References

[1] Torre LA, Bray F, Siegel RL, Ferlay J, Lortet-Tieulent J, Jemal A (2015). Global cancer statistics, 2012. CA Cancer J Clin.

[2] Moi P, Chan K, Asunis I, Cao A, Kan YW (1994). Isolation of NF-E2-related factor 2 (Nrf2), a NF-E2-like basic leucine zipper transcriptional activator that binds to the tandem NF-E2/AP1 repeat of the beta-globin locus control region. Proc Natl Acad Sci U S A.

[3] Itoh K, Chiba T, Takahashi S, Ishii T, Igarashi K, Katoh Y, Oyake T, Hayashi N, Satoh K, Hatayama I, Yamamoto M, Nabeshima Y (1997). An Nrf2/small Maf heterodimer mediates the induction of phase II detoxifying enzyme genes through antioxidant response elements. Biochem Biophys Res Commun.

[4] Venugopal R, Jaiswal AK (1996). Nrf1 and Nrf2 positively and c-Fos and Fra1 negatively regulate the human antioxidant response element-mediated expression of NAD(P)H:quinone oxidoreductase1 gene. Proc Natl Acad Sci U S A.

[5] Alam J, Stewart D, Touchard C, Boinapally S, Choi AM, Cook JL (1999). Nrf2, a Cap’n’Collar transcription factor, regulates induction of the heme oxygenase-1 gene. J Biol Chem.

[6] Jeyapaul J, Jaiswal AK (2000). Nrf2 and c-Jun regulation of antioxidant response element (ARE)-mediated expression and induction of gamma-glutamylcysteine synthetase heavy subunit gene. Biochem Pharmacol.

[7] Banning A, Deubel S, Kluth D, Zhou Z, Brigelius-Flohe R (2005). The GI-GPx gene is a target for Nrf2. Mol Cell Biol.

[8] Ishii T, Itoh K, Takahashi S, Sato H, Yanagawa T, Katoh Y, Bannai S, Yamamoto M (2000). Transcription factor Nrf2 coordinately regulates a group of oxidative stress-inducible genes in macrophages. J Biol Chem.

[9] Katoh Y, Itoh K, Yoshida E, Miyagishi M, Fukamizu A, Yamamoto M (2001). Two domains of Nrf2 cooperatively bind CBP, a CREB binding protein, and synergistically activate transcription. Genes Cells.

[10] Zhang DD (2006). Mechanistic studies of the Nrf2-Keap1 signaling pathway. Drug Metab Rev.

[11] Itoh K, Mimura J, Yamamoto M (2010). Discovery of the negative regulator of Nrf2, Keap1: a historical overview. Antioxid Redox Signal.

[12] Hayes JD, McMahon M, Chowdhry S, Dinkova-Kostova AT (2010). Cancer chemoprevention mechanisms mediated through the Keap1-Nrf2 pathway. Antioxid Redox Signal.

[13] Satoh H, Moriguchi T, Taguchi K, Takai J, Maher JM, Suzuki T, Winnard PT, Raman V, Ebina M, Nukiwa T, Yamamoto M (2010). Nrf2-deficiency creates a responsive microenvironment for metastasis to the lung. Carcinogenesis.

[14] Ramos-Gomez M, Kwak MK, Dolan PM, Itoh K, Yamamoto M, Talalay P, Kensler TW (2001). Sensitivity to carcinogenesis is increased and chemoprotective efficacy of enzyme inducers is lost in nrf2 transcription factor-deficient mice. Proc Natl Acad Sci U S A.

[15] Iida K, Itoh K, Kumagai Y, Oyasu R, Hattori K, Kawai K, Shimazui T, Akaza H, Yamamoto M (2004). Nrf2 is essential for the chemopreventive efficacy of oltipraz against urinary bladder carcinogenesis. Cancer Res.

[16] Aoki Y, Hashimoto AH, Amanuma K, Matsumoto M, Hiyoshi K, Takano H, Masumura K, Itoh K, Nohmi T, Yamamoto M (2007). Enhanced spontaneous and benzo(a)pyrene-induced mutations in the lung of Nrf2-deficient gpt delta mice. Cancer Res.

[17] Becks L, Prince M, Burson H, Christophe C, Broadway M, Itoh K, Yamamoto M, Mathis M, Orchard E, Shi R, McLarty J, Pruitt K, Zhang S, Kleiner-Hancock HE (2010). Aggressive mammary carcinoma progression in Nrf2 knockout mice treated with 7,12-dimethylbenz[a]anthracene. BMC Cancer.

[18] Kwak MK, Kensler TW (2010). Targeting NRF2 signaling for cancer chemoprevention. Toxicol Appl Pharmacol.

[19] Zhang Y, Gordon GB (2004). A strategy for cancer prevention: stimulation of the Nrf2-ARE signaling pathway. Mol Cancer Ther.

[20] Wang XJ, Sun Z, Villeneuve NF, Zhang S, Zhao F, Li Y, Chen W, Yi X, Zheng W, Wondrak GT, Wong PK, Zhang DD (2008). Nrf2 enhances resistance of cancer cells to chemotherapeutic drugs, the dark side of Nrf2. Carcinogenesis.

[21] Kensler TW, Wakabayashi N (2010). Nrf2: friend or foe for chemoprevention?. Carcinogenesis.

[22] Kawasaki Y, Okumura H, Uchikado Y, Kita Y, Sasaki K, Owaki T, Ishigami S, Natsugoe S (2014). Nrf2 is useful for predicting the effect of chemoradiation therapy on esophageal squamous cell carcinoma. Ann Surg Oncol.

[23] Curtis C, Shah SP, Chin SF, Turashvili G, Rueda OM, Dunning MJ, Speed D, Lynch AG, Samarajiwa S, Yuan Y, Graf S, Ha G, Haffari G, Bashashati A, Russell R, McKinney S, Langerod A, Green A, Provenzano E, Wishart G, Pinder S, Watson P, Markowetz F, Murphy L, Ellis I, Purushotham A, Borresen-Dale AL, Brenton JD, Tavare S, Caldas C, Aparicio S (2012). The genomic and transcriptomic architecture of 2,000 breast tumours reveals novel subgroups. Nature.

[24] McShane LM, Altman DG, Sauerbrei W, Taube SE, Gion M, Clark GM (2005). REporting recommendations for tumour MARKer prognostic studies (REMARK). Br J Cancer.

[25] Muller HM, Fiegl H, Goebel G, Hubalek MM, Widschwendter A, Muller-Holzner E, Marth C, Widschwendter M (2003). MeCP2 and MBD2 expression in human neoplastic and non-neoplastic breast tissue and its association with oestrogen receptor status. Br J Cancer.

[26] Cancer Genome Atlas Network. Comprehensive molecular portraits of human breast tumours. Nature. 2012; 490:61–70. 10.1038/nature11412 PMC3465532 23000897

[27] Bieche I, Franc B, Vidaud D, Vidaud M, Lidereau R (2001). Analyses of MYC, ERBB2, and CCND1 genes in benign and malignant thyroid follicular cell tumors by real-time polymerase chain reaction. Thyroid.

[28] Youden WJ (1950). Index for rating diagnostic tests. Cancer.

[29] Karihtala P, Kauppila S, Soini Y, Arja JV (2011). Oxidative stress and counteracting mechanisms in hormone receptor positive, triple-negative and basal-like breast carcinomas. BMC Cancer.

[30] Lau A, Villeneuve NF, Sun Z, Wong PK, Zhang DD (2008). Dual roles of Nrf2 in cancer. Pharmacol Res.

[31] Martin-Montalvo A, Villalba JM, Navas P, De CR (2011). NRF2, cancer and calorie restriction. Oncogene.

[32] Jaramillo MC, Zhang DD (2013). The emerging role of the Nrf2-Keap1 signaling pathway in cancer. Genes Dev.

[33] Sporn MB, Liby KT (2012). NRF2 and cancer: the good, the bad and the importance of context. Nat Rev Cancer.

[34] Moon EJ, Giaccia A (2015). Dual roles of NRF2 in tumor prevention and progression: possible implications in cancer treatment. Free Radic Biol Med.

[35] Kim YR, Oh JE, Kim MS, Kang MR, Park SW, Han JY, Eom HS, Yoo NJ, Lee SH (2010). Oncogenic NRF2 mutations in squamous cell carcinomas of oesophagus and skin. J Pathol.

[36] DeNicola GM, Karreth FA, Humpton TJ, Gopinathan A, Wei C, Frese K, Mangal D, Yu KH, Yeo CJ, Calhoun ES, Scrimieri F, Winter JM, Hruban RH, Iacobuzio-Donahue C, Kern SE, Blair IA, Tuveson DA (2011). Oncogene-induced Nrf2 transcription promotes ROS detoxification and tumorigenesis. Nature.

[37] Stacy DR, Ely K, Massion PP, Yarbrough WG, Hallahan DE, Sekhar KR, Freeman ML (2006). Increased expression of nuclear factor E2 p45-related factor 2 (NRF2) in head and neck squamous cell carcinomas. Head Neck.

[38] Homma S, Ishii Y, Morishima Y, Yamadori T, Matsuno Y, Haraguchi N, Kikuchi N, Satoh H, Sakamoto T, Hizawa N, Itoh K, Yamamoto M (2009). Nrf2 enhances cell proliferation and resistance to anticancer drugs in human lung cancer. Clin Cancer Res.

[39] Jiang T, Chen N, Zhao F, Wang XJ, Kong B, Zheng W, Zhang DD (2010). High levels of Nrf2 determine chemoresistance in type II endometrial cancer. Cancer Res.

[40] Konstantinopoulos PA, Spentzos D, Fountzilas E, Francoeur N, Sanisetty S, Grammatikos AP, Hecht JL, Cannistra SA (2011). Keap1 mutations and Nrf2 pathway activation in epithelial ovarian cancer. Cancer Res.

[41] Loignon M, Miao W, Hu L, Bier A, Bismar TA, Scrivens PJ, Mann K, Basik M, Bouchard A, Fiset PO, Batist Z, Batist G (2009). Cul3 overexpression depletes Nrf2 in breast cancer and is associated with sensitivity to carcinogens, to oxidative stress, and to chemotherapy. Mol Cancer Ther.

[42] Ren D, Villeneuve NF, Jiang T, Wu T, Lau A, Toppin HA, Zhang DD (2011). Brusatol enhances the efficacy of chemotherapy by inhibiting the Nrf2-mediated defense mechanism. Proc Natl Acad Sci U S A.

[43] Singh A, Boldin-Adamsky S, Thimmulappa RK, Rath SK, Ashush H, Coulter J, Blackford A, Goodman SN, Bunz F, Watson WH, Gabrielson E, Feinstein E, Biswal S (2008). RNAi-mediated silencing of nuclear factor erythroid-2-related factor 2 gene expression in non-small cell lung cancer inhibits tumor growth and increases efficacy of chemotherapy. Cancer Res.

[44] Solis LM, Behrens C, Dong W, Suraokar M, Ozburn NC, Moran CA, Corvalan AH, Biswal S, Swisher SG, Bekele BN, Minna JD, Stewart DJ, Wistuba II (2010). Nrf2 and Keap1 abnormalities in non-small cell lung carcinoma and association with clinicopathologic features. Clin Cancer Res.

[45] Yamadori T, Ishii Y, Homma S, Morishima Y, Kurishima K, Itoh K, Yamamoto M, Minami Y, Noguchi M, Hizawa N (2012). Molecular mechanisms for the regulation of Nrf2-mediated cell proliferation in non-small-cell lung cancers. Oncogene.

[46] Funes JM, Henderson S, Kaufman R, Flanagan JM, Robson M, Pedley B, Moncada S, Boshoff C (2014). Oncogenic transformation of mesenchymal stem cells decreases Nrf2 expression favoring in vivo tumor growth and poorer survival. Mol Cancer.

[47] Buffa FM, Camps C, Winchester L, Snell CE, Gee HE, Sheldon H, Taylor M, Harris AL, Ragoussis J (2011). microRNA-associated progression pathways and potential therapeutic targets identified by integrated mRNA and microRNA expression profiling in breast cancer. Cancer Res.

[48] Drafta D, Priscu A, Neacsu E, Gangura M, Schindler AE, Stroe E, Anghel C, Panaitescu G (1983). Estradiol and progesterone receptor levels in human breast cancer in relation to cytosol and plasma estrogen level. J Steroid Biochem.

[49] Okoh V, Deoraj A, Roy D (1815). Estrogen-induced reactive oxygen species-mediated signalings contribute to breast cancer. Biochim Biophys Acta.

[50] Wu J, Williams D, Walter GA, Thompson WE, Sidell N (2014). Estrogen increases Nrf2 activity through activation of the PI3K pathway in MCF-7 breast cancer cells. Exp Cell Res.

[51] Hartikainen JM, Tengstrom M, Kosma VM, Kinnula VL, Mannermaa A, Soini Y (2012). Genetic polymorphisms and protein expression of NRF2 and Sulfiredoxin predict survival outcomes in breast cancer. Cancer Res.

[52] Hartikainen JM, Tengstrom M, Winqvist R, Jukkola-Vuorinen A, Pylkas K, Kosma VM, Soini Y, Mannermaa A (2015). KEAP1 genetic polymorphisms associate with breast cancer risk and survival outcomes. Clin Cancer Res.

[53] Onodera Y, Motohashi H, Takagi K, Miki Y, Shibahara Y, Watanabe M, Ishida T, Hirakawa H, Sasano H, Yamamoto M, Suzuki T (2014). NRF2 immunolocalization in human breast cancer patients as a prognostic factor. Endocr Relat Cancer.

[54] Wakabayashi N, Slocum SL, Skoko JJ, Shin S, Kensler TW (2010). When NRF2 talks, who’s listening?. Antioxid Redox Signal.

